# Standardization of a CT Protocol for Imaging Patients with Suspected COVID-19—A RACOON Project

**DOI:** 10.3390/bioengineering11030207

**Published:** 2024-02-22

**Authors:** Andrea Steuwe, Benedikt Kamp, Saif Afat, Alena Akinina, Schekeb Aludin, Elif Gülsah Bas, Josephine Berger, Evelyn Bohrer, Alexander Brose, Susanne Martina Büttner, Constantin Ehrengut, Mirjam Gerwing, Sergio Grosu, Alexander Gussew, Felix Güttler, Andreas Heinrich, Petra Jiraskova, Christopher Kloth, Jonathan Kottlors, Marc-David Kuennemann, Christian Liska, Nora Lubina, Mathias Manzke, Felix G. Meinel, Hans-Jonas Meyer, Andreas Mittermeier, Thorsten Persigehl, Lars-Patrick Schmill, Manuel Steinhardt, The RACOON Study Group, Gerald Antoch, Birte Valentin

**Affiliations:** 1 Department of Diagnostic and Interventional Radiology, Medical Faculty and University Hospital, Heinrich Heine University Düsseldorf, 40225 Düsseldorf, Germany; 2 Department of Diagnostic and Interventional Radiology, Eberhard Karls University Tuebingen, Hoppe-Seyler-Strasse 3, 72076 Tuebingen, Germanyjosephine.berger@med.uni-tuebingen.de (J.B.); 3 Clinic and Outpatient Clinic for Radiology, University Hospital Halle (Saale), 06120 Halle, Germanyalexander.gussew@uk-halle.de (A.G.); 4 Department of Radiology and Neuroradiology, University Hospital Schleswig-Holstein Campus Kiel, 24105 Kiel, Germany; 5 Department of Diagnostic and Interventional Radiology, University Hospital of Marburg, 35043 Marburg, Germany; 6 Department of Diagnostic and Interventional Radiology, University Hospital Giessen, Justus Liebig University, Klinikstr. 33, 35392 Giessen, Germany; 7 Department of Diagnostic and Interventional Radiology, Ulm University Medical Center, Albert-Einstein-Allee 23, 89081 Ulm, Germanychristopher.kloth@uniklinik-ulm.de (C.K.); 8 Department of Diagnostic and Interventional Radiology, University of Leipzig Medical Center, Liebigstraße 20, 04103 Leipzig, Germany; 9 Clinic of Radiology, University of Münster, 48149 Münster, Germany; 10 Department of Radiology, LMU University Hospital, LMU Munich, 81377 Munich, Germany; 11 Department of Radiology, Jena University Hospital, Friedrich Schiller University, 07747 Jena, Germanyandreas.heinrich@med.uni-jena.de (A.H.); 12 Institute of Diagnostic and Interventional Radiology, School of Medicine and Health, Technical University of Munich, 81675 Munich, Germany; 13 Institute for Diagnostic and Interventional Radiology, Faculty of Medicine and University Hospital Cologne, University of Cologne, 50937 Cologne, Germany; 14 Department of Diagnostic and Interventional Radiology and Neuroradiology, University Hospital Augsburg, Stenglinstraße 2, 86156 Augsburg, Germany; 15 Institute of Diagnostic and Interventional Radiology, Paediatric Radiology and Neuroradiology, University Medical Centre Rostock, Schillingallee 36, 18057 Rostock, Germany

**Keywords:** COVID-19, computed tomography, image quality, radiation exposure

## Abstract

CT protocols that diagnose COVID-19 vary in regard to the associated radiation exposure and the desired image quality (IQ). This study aims to evaluate CT protocols of hospitals participating in the RACOON (Radiological Cooperative Network) project, consolidating CT protocols to provide recommendations and strategies for future pandemics. In this retrospective study, CT acquisitions of COVID-19 patients scanned between March 2020 and October 2020 (RACOON phase 1) were included, and all non-contrast protocols were evaluated. For this purpose, CT protocol parameters, IQ ratings, radiation exposure (CTDI_vol_), and central patient diameters were sampled. Eventually, the data from 14 sites and 534 CT acquisitions were analyzed. IQ was rated good for 81% of the evaluated examinations. Motion, beam-hardening artefacts, or image noise were reasons for a suboptimal IQ. The tube potential ranged between 80 and 140 kV_p_, with the majority between 100 and 120 kV_p_. CTDI_vol_ was 3.7 ± 3.4 mGy. Most healthcare facilities included did not have a specific non-contrast CT protocol. Furthermore, CT protocols for chest imaging varied in their settings and radiation exposure. In future, it will be necessary to make recommendations regarding the required IQ and protocol parameters for the majority of CT scanners to enable comparable IQ as well as radiation exposure for different sites but identical diagnostic questions.

## 1. Introduction

Several CT protocols for diagnosing and monitoring the course of COVID-19 and its treatment have been published since the beginning of the pandemic for a variety of CT scanners and vendors [1,2,3,4,5,6,7,8]. Quite often, protocols vary with regard to the associated radiation exposure and the desired image quality (IQ). There are already few reviews and evaluations published that compare different protocols with regard to the sensitivity and specificity of the diagnosis of COVID-19 [9,10,11,12,13]. Discrepancies in CT protocols for a particular organ or diagnostic inquiry are frequently observed within a single hospital, which is contingent upon the quantity of CT scanners and the diversity of vendors involved. At present, there is no established or officially defined threshold for minimum or maximum IQ for evaluating COVID-19.

Additionally, the preferred IQ partially relies on the interpretation skills of the reader. Nevertheless, variations in CT protocol parameter settings can significantly impact IQ, posing potential challenges. Problems can arise when patients are transferred between different hospitals or when they receive a follow-up scan months later at a different hospital to investigate any post-infectious parenchymal alterations related to long COVID [14,15].

In the majority of examinations to evaluate COVID-19, contrast agents have not been administered. Nonetheless, the application of contrast agents becomes necessary in certain patients to assess emergency situations or disease-related secondary manifestations, such as pulmonary artery embolism. These protocols need to fulfill different criteria compared to non-enhanced CT acquisitions. Typically, contrast-enhanced acquisitions entail a higher level of radiation exposure [5].

In general, there is a need for minimal radiation exposure while simultaneously ensuring adequate IQ for highly reliable diagnosis, exclusion of COVID-19, or to rule out incidental findings. The radiation exposure of CT acquisitions is subject to national diagnostic reference levels. These levels for chest CT, independent of contrast agent administration, vary between 6 mGy and 10 mGy in Germany, the United Kingdom, Australia, Belgium, and Canada [16,17,18,19,20,21]. The radiation exposure should remain within the reference levels unless there are compelling reasons not to do so (e.g., obese patients, challenging patient positioning). Although the majority of patients with more severe infections are older in age, young and even pediatric patients also require CT scans for diagnosis and treatment supervision [22,23].

Various established criteria for diagnosing COVID-19, including the Fleischner criteria [24] and recommendations from organizations like the American College of Radiology (ACR), European Society of Radiology (ESR), Radiological Society of North America (RSNA), German Radiological Society (DRG), or World Health Organization (WHO) have been published [25,26,27,28,29]. Nonetheless, these guidelines primarily address the selection of imaging modalities (e.g., X-ray, CT, and ultrasound) in relation to clinical factors and the approach to image interpretation. However, they do not supply detailed CT protocol specifications.

The quantitative imaging biomarkers alliance (QIBA) of the RSNA provides recommendations for COVID-19 CT protocols [30]. They recommend starting the acquisition at the position where movement is strongest and finish the acquisition within five seconds [30]. Protocols should include the use of automatic exposure control to adapt the radiation exposure. In general, the volumetric CT dose index (CTDI_vol_) of these scans should remain within 3 mGy for average-sized patients. To reduce the radiation exposure while maintaining sufficient IQ, iterative reconstruction is recommended. Reconstructed slice thickness should be ≤1.25 mm with overlapping slices [30]. Kwee et al. published requirements for CT imaging in COVID-19 diagnosis [31]. Usually, a non-contrast agent-enhanced CT should be performed during a single inspiratory breath-hold with an LD protocol to reduce the radiation exposure. For this purpose, several techniques should be employed, such as a low tube potential, spectral shaping of the X-ray beam using a tin filter (if available), and a high pitch and short rotation time to reduce motion artefacts. During post-processing, iterative reconstruction or deep learning methods for noise reduction should be applied. To enable detailed lung structure evaluation, a sharp kernel is recommended [31]. 

The national guidelines for chest CT in general differ between non-enhanced, low-dose (LD), and high-contrast CT and full-dose (FD) CT, with or without contrast enhancement [32]. Variations can be identified by the required level of image detail that must be perceptible, e.g., LD ≤ 2 mm for high contrast and ≤ 4 mm for low contrast vs. FD ≤ 1 mm high contrast and ≤2 mm low contrast. Further requirements are the tube potential settings between 100 kV_p_ and 130 kV_p_ for LDCT and 80 kV_p_ and 120 kV_p_ for FDCT, a detector collimation < 1 mm for LDCT and <0.75 mm for FDCT, and a spiral pitch factor between 0.9 and 1.2. Specific requirements for CTDI_vol_ are not provided, except that the diagnostic reference values should be adhered to [32].

The German Commission on Radiological Protection (“Strahlenschutzkommission” (SSK)) recommends in their report from 2021 the usage of an LDCT for the diagnosis of COVID-19 only after justifying an indication with a CTDI_vol_ < 3 mGy for patients with slim or normal stature [33]. They do, however, acknowledge that lower level of radiation exposure might be achievable. Kalra et al. also recommend maximum radiation exposure in terms of the CTDI_vol_-value < 3 mGy for small- or average-sized patients [5]. 

While the COVID-19 pandemic is currently less prominent, there is a possibility that new epidemics or pandemics will emerge in the future. In such scenarios, the fast establishment of standardized imaging techniques would be valuable.

Within the German Radiological Cooperative Network (RACOON, https://racoon.network/, accessed on 19 February 2024), clinical and radiological data of non-enhanced COVID-19 CT acquisitions of several university hospitals in Germany have been collected and processed for evaluation. This study aims to evaluate the CT protocols of hospitals participating in the RACOON project, consolidating CT protocols to provide recommendations and strategies for future pandemics or studies with collaborating hospitals.

## 2. Materials and Methods

All institutional review boards of participating sites approved this retrospective study for the individual sites. RACOON sites willing to participate sent their anonymized protocol and exposure settings for CT acquisitions of patients with a confirmed diagnosis of COVID-19. All patients were part of RACOON phase 1, which ranged from March 2020 to October 2020.

### 2.1. Data Collection

All patients within the COVID-19 cohort of the RACOON dataset had a confirmed positive COVID-19 test at the time of their CT examination. The pre-specified major findings and subjective IQ were documented. At each site, IQ was rated in consensus by a (senior) assistant and a board-certified radiologist. IQ was rated suboptimal or insufficient if one or a combination of the following factors was present: image noise, motion or beam-hardening artefacts, or an insufficient field of view. If the IQ was so poor that it was no longer possible to make a confidential diagnosis, the IQ was classified as insufficient. Image noise is usually a protocol-specific problem. Here, a too low tube current might have caused increased image noise or an insufficient noise reduction during image post-processing. Motion or beam-hardening artefacts are typically caused by the patient due to breathing motion or restless behavior. An insufficient field is either caused by off-center positioning of the patient or a cropped reconstructed field of view by the technician.

**Figure 1:**
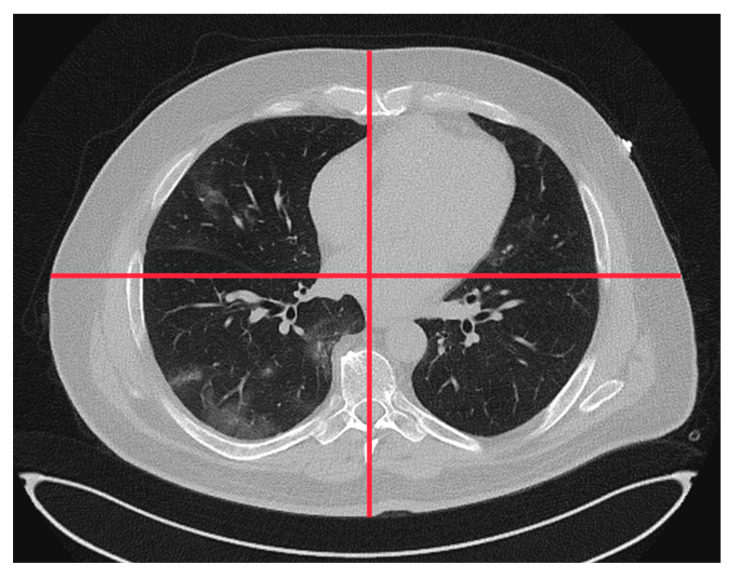
Central anterior–posterior and lateral diameter measured on the CT images (lung window) at the height of the heart.

The requested patient and scanner data, major findings, and subjective IQ parameters from the participating RACOON sites are provided in Tables S1 and S2 in the Supplementary Materials. Data from the different sites were either sampled manually or collected using a site’s dose management system. Patient anterior–posterior (D_ap)_ and lateral diameter (D_lat_) were measured at the level of the heart (see Figure 1). 

The scan length was either provided by a dose management system or calculated using the dose length product (DLP) and CTDI_vol_. To ensure that the measurements were comparable, we utilized the following formula to compute scan length for all sites:

(1) $$Scan\;length\;\left[mm\right]=10\times \frac{DLP\;[mGy\cdot cm]}{{CTDI}_{vol}\;[mGy]},$$

Furthermore, the size-specific dose estimate (SSDE) was calculated for each acquisition using the measured diameters and the CTDI_vol_ using the effective diameter-specific conversion coefficients according to the AAPM report no. 204 [34].

(2) $$Effective\;diameter\;\left[cm\right]=\sqrt{D_{ap\;}\left[cm\right]\times D_{lat}\;[cm]}$$

(3) $$SSDE\;\left[mGy\right]=conversion\;coefficient\times {CTDI}_{vol}\;[mGy]$$

### 2.2. Exclusion Parameters

Due to the variety of indications, requirements, and protocols for contrast-enhanced CT acquisitions, only non-enhanced (native) chest CT acquisitions were evaluated in this study. Acquisitions with scan lengths < 20 cm and >60 cm were excluded from the analysis since this study focused primarily on chest CT acquisitions. Acquisitions without radiation exposure data were excluded.

### 2.3. Site-Specific Evaluation

For each site, the radiation exposure parameters (CTDI_vol_ and DLP), scan range, subjective IQ (three-point scale: good, suboptimal, and insufficient IQ), and patient anterior–posterior, lateral, and effective diameter were evaluated based on the following subgroups:

1. Analysis A: Site-specific evaluation of the exposure parameters and image quality.

For all scanners and sites, all non-enhanced CT acquisitions were evaluated with regard to the above-mentioned parameters.

2. Analysis B: Scanner-specific protocol analysis for non-enhanced CT examinations with ≥5 examinations per protocol per site.

Protocols with ≥5 acquisitions per protocol were evaluated, based on the chosen tube potential, tube current–time product (TCTP), collimation, spiral pitch factor, and rotation time. For this purpose, all protocols were evaluated with identical pitch, rotation time, collimation, and reference exposure values (either tube potential and TCTP value or noise index (NI), which were more than five times employed). The mean CTDI_vol_ for each protocol was provided for these examinations.

Data were consolidated using Excel (Microsoft Office, version 2021). The R software (v4.3.2, R Foundation for Statistical Computing, Vienna, Austria) was used for visualization of the data. 

## 3. Results

### 3.1. Site-Employed CT Scanners

In total, 15 German university radiology departments participated in this study. A variety of scanners (vendors and models) were available, as described in Table S3. The majority of sites used single-energy scanners (see Table 1); these also included scanners with the possibility of dual-energy imaging. However, only one dual-energy scan was performed throughout the cohort. Furthermore, two sites used dual-source CT examinations but with only one single tube activation.

The number of included patients varied considerably per site (4–105 non-enhanced examinations). Only one site provided examinations with contrast-enhanced acquisitions. The data from this site could therefore not be evaluated for the purpose of this study.

### 3.2. Analysis A: Site-Specific Evaluation of the Exposure Parameters and Image Quality

**Figure 2:**
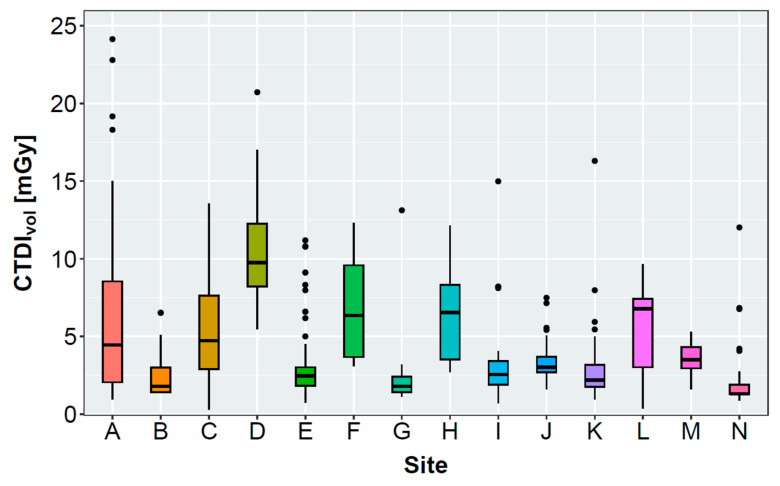

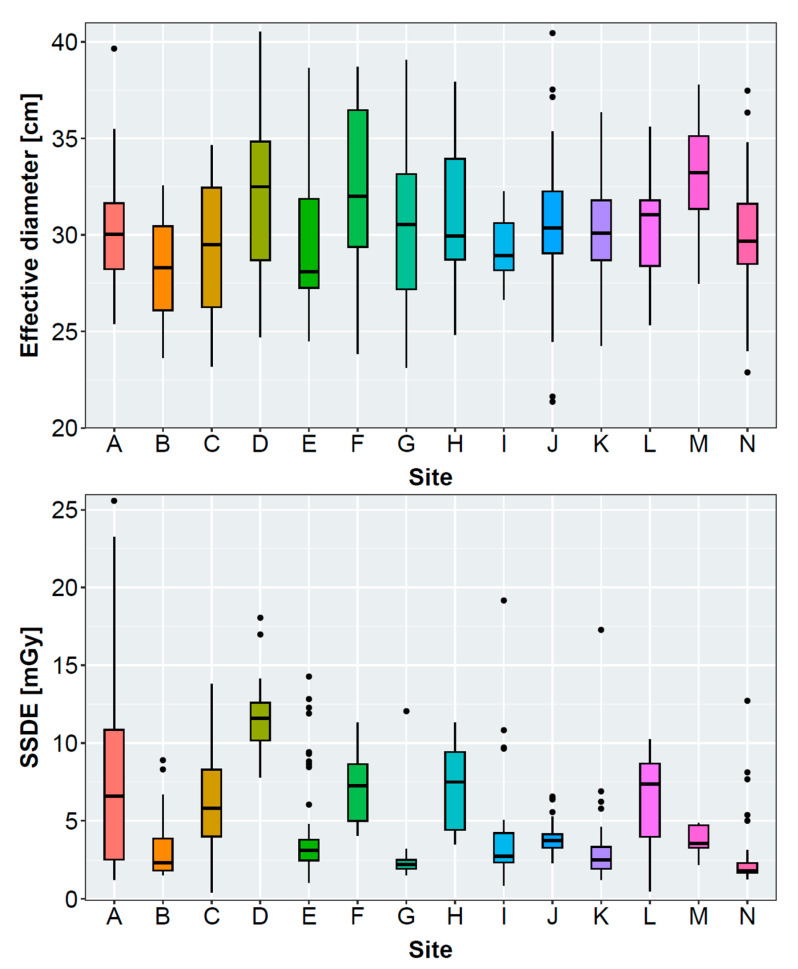
Boxplot graphs presenting the volumetric computed tomography dose index (CTDI_vol_), effective diameter, and size-specific dose equivalent (SSDE) of the evaluated sites. Effective diameters and SSDE were not measurable for 2/37 (site B), 5/30 (site I), and 22/64 (site K) patients due to restricted reconstructed field of view.

Table 2 and Figure 2 present the results of the evaluated radiation exposure parameters, patient diameter, scan length, and subjective IQ of all included non-enhanced acquisitions per site. Mean ap-diameters ranged between 23.9 ± 2.8 cm and 28.4 ± 5.4 cm. Mean lateral diameters ranged between 33.7 ± 3.5 cm and 39.3 ± 4.0 cm, with a maximum difference of approximately 5 cm between the sites. Mean CTDI_vol_ ranged between 2.0 ± 1.7 mGy and 10.7 ± 3.9 mGy. The DLP also varied considerably between 67.0 ± 63.1 mGycm and 323.2 ± 112.9 mGycm. The scan length ranged between 303.9 ± 34.9 mm and 422.3 ± 26.6 mm. 

The IQ of the majority of acquisitions was rated “good” or “suboptimal”. When available, the assessment included an evaluation of IQ and an investigation into the causes of suboptimal or inadequate IQ. In total, the IQ of 433/534 (81%) examinations was evaluated. IQ was rated “good” in 350/433 (81%), “suboptimal” in 81/433 (19%), and “insufficient” in 2/433 (<1%) of the examinations. For 82/83 of the suboptimal and insufficient rated examinations, reasons were documented. Inadequate IQ in examinations was caused by motion artefacts (62/82, 76%), beam-hardening artefacts (4/82, 5%), image noise (2/82, 2%), insufficient FOV (2/82, 2%), or a combination of these factors, see Table 3.

### 3.3. Analysis B: Scanner-Specific Protocol Analysis for Non-Enhanced CT Examinations with ≥5 Examinations per Protocol per Site

Several varying protocols were available from the different sites (see Table 4 and Table S4). Only some sites used one or two specific protocols for non-enhanced CT acquisitions, whereas other sites used a variety of CT protocols. Those were adapted to patient habitus, indication, or CT scanner specifications.

The reference tube potential ranged between 100 kV_p_ and 120 kV_p_ for the obtained CT protocols. The reference values for TCTP (TCTP_ref_) ranged between 20 and 110 mAs for acquisitions at 120 kV_p_, between 19 and 51 mAs at 110 kV_p_, and between 60 and 124 mAs at 100 kV_p_. Two protocols employed a fixed TCTP of 20 mAs at 120 kV_p_. For scanners employing an NI, the NI ranged between 23 and 35. At one site, examinations were performed in the dual-source mode (100–140 kV_p_) with a pitch of 1.7. In most of the protocols, the effective TCTP (TCTP_eff_) was higher than the TCTP_ref_ (see Table S4). Most patients with higher TCTP_eff_ values than TCTP_ref_ had a higher effective diameter than patients with lower TCTP_eff_ values than TCTP_ref_ (see Table S4).

The total detector collimation width ranged between 12 mm and 80 mm. The single collimation width ranged between 0.60 mm and 1.25 mm. The rotation time throughout the whole cohort ranged between 0.23 s and 0.70 s. However, in the majority of the examinations, the rotation time was shorter than 0.5 s. The rotation time is kept short to reduce the artefacts resulting from the movement of the heart. A high rotation time can lead to motion artefacts due to the heartbeat and/or if breath-holding is insufficient.

All acquisitions were performed using helical acquisitions. The spiral pitch factor ranged between 0.55 and 1.40. However, in the majority of examinations, a spiral pitch factor between 0.8 and 1.2 was chosen.

Corresponding average CTDI_vol_-values ranged between 1.4 and 10.7 mGy for the provided protocols, with the majority of protocols < 5 mGy.

## 4. Discussion

In the context of COVID-19 patients, it was observed that the majority of the healthcare facilities included in this study did not have a dedicated low-dose protocol for pulmonary infections or had valid reasons for not applying a low-dose protocol. Throughout the participating sites, a large number of different CT scanners and vendors were employed. CT scanners varied in terms of age and specifications, influencing the required radiation exposure to obtain a sufficient image signal. This variety also implied different scanning and evaluation methods. This observation has been previously documented in other countries [35]. Sites employing several protocols explained that the need for different indications necessitated distinct protocols. However, especially in cases where different CT scanners were in use, protocols were adapted to the available specifications of the CT scanners. Only few sites had a dedicated CT scanner where COVID-19 patients were predominantly examined with a specific protocol.

During their COVID-19 treatment, patients were transferred between hospitals if medical care required this [15]. In these types of situations, CT protocol standardization will help ensure a proper imaging follow-up and will finally help to prevent repeated examinations, reducing the cost of the treatment. During the pandemic, collaborations such as the RACOON consortium were founded, helping to harmonize imaging and image reading between university hospitals. As Coccia et al. describe, governments have to plan policies of public health to cope with future infections [36]. For this purpose, international collaborations in research and healthcare are required. The implementation of these collaborations is complex; hence, long-term preparation is required [36].

### 4.1. Radiation Exposure at the Different Sites

The employed radiation exposure varied considerably between the different sites. Still, all sites have to stick to the national diagnostic reference levels [20]. The majority of sites had mean CTDI_vol_-values lower than the diagnostic reference levels of chest CT examinations in the year 2020 (CTDI_vol_ = 10 mGy; DLP = 350 mGycm). All mean DLPs were well within the reference level. Even with the updated diagnostic reference levels (CTDI_vol_ = 8 mGy for chest CT [20]), which were not yet valid at the time of the study, all except one site remained within the new upper reference level. The German DRL do not differ between contrast-enhanced and non-enhanced acquisitions. Still, one would tend to expect a slightly lower dose for non-enhanced acquisitions and hence, a more clearly undercutting of the DRL. The variety in CTDI_vol_ values that were collected shows the different use of low- or normal-dose protocols at the different sites. 

After evaluating all non-enhanced acquisitions, only 5 of 14 sites reached a mean CTDI_vol_ below or equal to the recommended 3 mGy from the SSK (see Table 2) [33]. Reasons for this might be a tendency toward overweight or obese patients being examined in the study cohort. Figure 2, Table 4, and Table S4 show that a high CTDI_vol_ was often accompanied with a high effective diameter for sites that employ a tube current modulation. Here, the CT scanner increases the tube current for overweight or obese patients to maintain sufficient IQ. Sites D, H, and F examined patients with large effective diameters, requiring a severe increase in the radiation exposure. 

From the evaluated individual protocols (Table 4), approximately 50% were within the recommendations of the SSK. Some of these protocols were specifically designed for image pulmonary infections, e.g., COVID-19, employing considerably less radiation than FD protocols. No protocol yielded a mean CTDI_vol_ value below 1 mGy in comparison to other published protocols [1,13,37,38,39]. In some published studies, patients received an FD and an LD (CTDI_vol_ < 1 mGy) CT examination directly one after the other. Afterwards, imaging findings, IQ, and radiation exposure were compared [1,37,39]. The LD examinations had a lower objective and subjective IQ, which can be increased using post-processing methods. A disadvantage of post-processing methods is the risk of altering the image data such that small details might be removed and not be perceptible anymore [1]. These LD examinations (CTDI_vol_ < 1 mGy) might be employed for follow-up examinations; however, for new and confident diagnoses, the IQ might not be sufficient. However, within the clinical introduction of photon-counting CT scanners and post-processing algorithms, low-dose CT scans with acceptable quality might be achievable in the future [40].

### 4.2. Image Quality

The IQ of the majority of CT images was rated “good”. In this study, a good or adequate IQ is not specifically described in terms of the contrast-to-noise ratio (CNR) or signal-to-noise ratio (SNR), but it depends on the image impression in general. If the image noise, motion, or beam-hardening artefacts have too strong an influence on the imaging, the overall IQ was rated lower. The majority of images with suboptimal IQ resulted from motion artefacts, which are usually protocol-independent (if the rotation time is not exceeding a rotation time of 0.5 s),which tend to be caused by patients. Only 2% of the suboptimal image quality was solely caused by image noise, which suggests that the underlying protocol settings led to a sufficient IQ in the vast majority of patients. A certain number of patients could not react to breathing commands or be positioned ideally in the CT scanner, resulting in a suboptimal IQ. Severely diseased and/or sedated patients are not able to hold their breath. Breathing and patient positioning can cause motion and/or beam-hardening artefacts, thereby increasing image noise and deteriorating IQ. 

### 4.3. Protocol Settings

The tube potential and TCTP influence the radiation exposure considerably. The protocols with the lowest radiation exposure values in this study employed TCTP values of 19–20 mAs at 110–120 kV_p_, resulting in CTDI_vol_ values of approximately 1.4 mGy. As described above, there were protocols published with CTDI_vol_ values well below 1 mGy with TCTPs as low as 10 mAs at 100 kV_p_ or 180 mAs at tin (Sn)-filtered 100 kV_p_ on modern but widely used CT scanners [37,39]. However, these LD examinations were parts of feasibility studies rather than being the sole diagnostic examination.

A reference tube potential of 100 kV_p_ (with or without tin filter) should be a reasonable compromise between IQ and radiation exposure. The NI, as required by GE scanners, is difficult to compare to the tube potential and TCTP values. A large number (26) of CT scanners of different ages and vendors were used within this study. Among the evaluated protocols (Table 4), there were two CT scanners running on different sites with different protocols, reaching considerably different CTDI_vol_-values (e.g., site D and I). Depending on the chosen TCTP_ref_, the resulting radiation exposure is altered. Unfortunately, we did not evaluate the use of iteratives of deep learning reconstruction methods. With iterative reconstruction methods, it is possible to allow a dose reduction of up to 50% in chest imaging [41]. However, the use of different reconstruction techniques alone does not explain the differences in the chosen TCTP_ref_.

The rotation time and spiral pitch factor influence the duration of the acquisitions. Typically, chest acquisitions are performed with breath-hold techniques. Newer CT scanners allow for rotation times ≤ 0.285 s, reducing the occurrence of motion/breathing artefacts. Hence, a fast rotation time is advantageous to increase the IQ. The majority of published protocols employ pitch values close to or larger than one [7,39,42]. This is in accordance with our results. However, under certain circumstances, the spiral pitch factor cannot be modified. For example, the activation of the modulation type “X-CARE” for Siemens CT scanners constrains the spiral pitch factor to exactly 0.6.

The detector collimation width differed considerably between the evaluated sites (between 12 and 80 mm). A large detector coverage can allow for lesser required tube rotations to obtain the complete scan coverage. This, again, reduces motion artefacts. In contrary, a smaller detector collimation requires longer scanning times, which increases the incidence of motion artefacts. Usually, the largest detector collimation width is employed to increase the imaged volume during one rotation. Some CT scanners offer a smaller detector element size in the center of the detector (e.g., 16 rows of 0.3 mm detector elements instead of 0.6 mm elements). The smaller detector collimation can increase image resolution and is advantageous in some diagnostic questions. However, none of the evaluated sites employed a single detector element width < 0.6 mm. In the literature, only one publication was found with a smaller detector collimation of 120 × 0.2 mm using a photon-counting CT (PCT) [43]. PCTs were not released before 2021; hence, they were released after the time of image acquisition for this study. Since this technique is not available at most sites, a detector collimation of ≤ 0.6 mm with the advantage of a fast image acquisition is recommended to enable all hospitals to actually implement those recommended settings. A decrease in the collimation width can enable thinner slices for image diagnosis, but unfortunately, this requires a higher tube current to maintain a sufficient signal-to-noise ratio level. If not compensated otherwise (e.g., by new reconstruction techniques), the radiation exposure might increase considerably.

### 4.4. Limitations

A number of limitations of the presented study need to be mentioned. First of all, the comparability between different CT scanners is difficult. The way that the automatic exposure control is implemented differs between vendors. Hence, it is primarily possible to propose expected exposure values (in terms of CTDI_vol_) and scan parameters (pitch, collimation, and tube rotation). The final implementation on the CT scanner needs to be adjusted to reach these values. The availability of acquisition data varied considerably. On the one hand, the number of acquisitions varied. On the other hand, data processing depended on the available resources at the sites (the use of dose management systems vs. the manual recording of data). Furthermore, post-processing of image data was not evaluated in this study. With modern CT scanners, several post-processing methods are available, either improving IQ or reducing the employed radiation exposure. It is assumed that most of the sites in this study cohort have a post-processing method available and employed (either iterative reconstruction or a deep learning algorithm). Furthermore, image data could not be evaluated objectively since the data were not sharable for this analysis due to ethical and data privacy protection restrictions. Unfortunately, not all lateral diameters were available due to restricted field of views in the reconstructions. For one site, the image quality rating was not available for nearly all of the examinations. We marked lacking diameters and lacking image quality ratings in the tables and figures. 

There is a bias in this study, as patients in university hospitals often have a more severe clinical picture. In general, however, protocols should be applicable across a large population and disease levels. We assume that the recommendations shown here are generally applicable for adult patients on the one hand, in relation to the population, and on the other hand, in relation to CT devices.

## 5. Conclusions

This study demonstrates the urgency of standardized CT protocols in a pandemic. Both standardized methods for image acquisition and image reading are necessary and should be implementable at different sites. Consolidated protocol settings enable comparable IQ as well as radiation exposure for different sites with the same diagnostic questions. The developed protocol needs to fulfil the requirements of the national health authorities. Patients still might need to undergo specified CT examinations if comorbidities or the medical condition requires so (e.g., administration of contrast agents). In these cases, a standardized protocol might not be applicable.

In the future, it will be necessary not only to make recommendations as to when examination should take place but also to make recommendations regarding the required IQ and protocol parameters for the majority of CT scanners. For future pandemics, it is important to quickly generate CT protocols that are easily accessible and applicable on different CT scanners to ensure comparable IQ and radiation exposure for many sites. Especially under demanding conditions during a pandemic, the many patients with the same new diagnostic questions who need to receive a CT scan within a short period of time can benefit from an optimized and consolidated CT protocol. 

Moreover, for imaging feature extraction and data analysis to detect clinically important imaging features, homogenous and robust CT quality and protocols are desired. 

## Figures and Tables

**Table 1:** Number of included examinations, CT scanner types, acquisition mode, and contrast-enhancement throughout the cohort.

Site	# Examinations Included	# CT Scanner Types *	Scan Mode (SE/DE/DS)
A	51	6	SE (51)
B	37	3	SE (37)
C	4	3	SE (3), DS (1)
D	20	1	SE (20)
E	105	5	SE (80), DS (25)
F	10	2	SE (10)
G	49	1	SE (49)
H	20	1	SE (20)
I	30	2	SE (30)
J	61	2	SE (61)
K	64	4	SE (64)
L	17	2	SE (17)
M	7	3	SE (7)
N	59	2	SE (58), DE (1)
O	only contrast-enhanced CT scans, therefore excluded		

SE = single-energy; DE = dual-energy; DS = dual-source. * More than one CT scanner of each type possible.

**Table 2:** Site-specific evaluation of all included non-enhanced CT acquisitions per site, independent of the chosen protocol. Data are provided as mean ± standard deviation for the diameters, CTDI_vol_, SSDE, DLP, and scan length. For the image quality, the numbers of acquisitions per rating scale were provided.

Site	n	Diameter (cm)		Effective Diameter (cm)	CTDI_vol_ (mGy)	SSDE (mGy)	DLP (mGycm)	Calculated Scan Length (mm)	Image Quality Good/Suboptimal/ Not Sufficient/n.e.
		ap	lat						
A	51	25.6 ± 2.9	35.6 ± 3.7	30.2 ± 3.0	6.4 ± 5.6	7.4 ± 5.8	246.4 ± 252.2	368.5 ± 45.5	41/9/0/1
B	37	23.9 ± 2.8	33.7 ± 3.5 ^a^	28.1 ± 2.6 ^a^	2.5 ± 1.5	3.2 ± 1.9 ^a^	79.0 ± 47.5	323.9 ± 39.6	20/17/0/0
C	4	24.9 ± 4.4	34.3 ± 6.1	29.2 ± 5.0	5.8 ± 5.6	6.5 ± 5.5	202.8 ± 197.1	337.9 ± 26.8	4/0/0/0
D	20	27.2 ± 4.5	37.6 ± 5.1	31.9 ± 4.5	10.7 ± 3.9	11.8 ± 2.6	323.2 ± 112.9	304.5 ± 24.3	17/3/0/0
E	105	25.3 ± 3.7	34.5 ± 4.2	29.5 ± 3.5	3.0 ± 2.1	3.7 ± 2.4	92.1 ± 71.6	303.9 ± 34.9	6/0/0/99
F	10	28.4 ± 5.4	37.0 ± 4.3	32.3 ± 4.9	6.8 ± 3.3	7.1 ± 2.4	286.1 ± 139.5	422.3 ± 26.6	9/1/0/0
G	49	25.1 ± 3.3	37.4 ± 5.0	30.6 ± 3.7	2.1 ± 1.7	2.4 ± 1.5	85.7 ± 68.4	399.1 ± 35.3	45/3/0/1
H	20	25.2 ± 3.3	38.5 ± 5.1	31.1 ± 3.8	6.5 ± 3.4	7.2 ± 2.8	247.7 ± 130.8	381.0 ± 21.6	20/0/0/0
I	30	24.0 ± 1.8	35.7 ± 2.2 ^b^	29.3 ± 1.7 ^b^	3.4 ± 2.9	4.3 ± 3.9 ^b^	110.2 ± 98.5	327.2 ± 35.5	24/6/0/0
J	61	25.3 ± 3.5	37.2 ± 5.3	30.5 ± 3.4	3.3 ± 1.1	3.9 ± 0.9	103.2 ± 36.7	314.9 ± 30.1	58/3/0/0
K	64	25.7 ± 2.6	35.7 ± 3.7 ^c^	30.4 ± 2.6 ^c^	2.8 ± 2.2	3.2 ± 2.6 ^c^	93.5 ± 104.3	323.3 ± 47.5	38/24/2/0
L	17	25.9 ± 3.0	36.2 ± 2.9	30.6 ± 2.7	5.4 ± 2.8	6.3 ± 3.0	181.4 ± 95.8	339.7 ± 40.3	10/7/0/0
M	7	27.9 ± 3.1	39.3 ± 4.0	33.1 ± 3.4	3.6 ± 1.2	3.8 ± 1.0	133.4 ± 54.4	370.7 ± 37.0	4/3/0/0
N	59	25.8 ± 2.9	34.7 ± 3.8	29.9 ± 3.0	2.0 ± 1.7	2.4 ± 1.9	67.0 ± 63.1	330.8 ± 41.2	54/5/0/0
All	534	25.4 ± 3.3	35.8 ± 4.4	30.1 ± 3.4	3.7 ± 3.4	4.4 ± 3.6	127.4 ± 130.4	335.5 ± 49.2	350/81/2/101

n.e. = not evaluated; CTDI_vol_ = volumetric computed tomography dose index; SSDE = size-specific dose equivalent; DLP = dose length product. Diameters not measured for ^a^ 2 (site B), ^b^ 5 (site I), and ^c^ 22 (site K) patients due to restricted reconstructed field of view.

**Table 3:** Reasons for suboptimal or insufficient image quality.

Reason for Suboptimal or Insufficient Image Quality	# Acquisitions with Provided Reason for Suboptimal or Insufficient Image Quality, n = 82	Percentage Acquisitions per Reason (%)
Motion	62	76
Beam hardening	4	5
Image noise	2	2
Insufficient FOV	2	2
Motion, beam hardening, andimage noise	2	2
Motion and insufficient FOV	3	4
Motion and beam hardening	4	5
Motion and image noise	3	4

FOV = field of view.

**Table 4:** Employed protocols (≥5 performed acquisitions) with CT scanner settings and mean CTDI_vol_ for the included patients. Protocols were sorted based on the reference tube potential and reference tube current–time product (TCTP). Corresponding anterior–posterior and lateral diameters can be found in Table S4.

Site (Included Patients)	Ref. Tube Potential (kV)	Reference TCTP (mAs) or *NI*	Pitch	Rotation Time (s)	Total Collimation (Single Detector Element Size) (mm)	Effective Diameter with Range (cm)	Mean CTDI_vol_ with Range (mGy)	Mean SSDE with Range (mGy)	IQ Good/ Suboptimal/ Not Sufficient/n.e.
D (20)	120	110	1.1	0.5	19.2	31.9 (24.7–40.5)	10.7 (5.5–20.7)	11.8 (7.8–18.0)	17/3/0/0
B (11)	120	58	0.763	0.33	80.0 (0.625)	28.0 (23.6–32.3)	3.7 (2.3–6.5)	4.8 (3.0–8.9)	7/4/0/0
G (48)	120	15–50 (29) ^+^	0.763	0.33	80.0 (0.625)	30.5 (23.1–39.1)	1.9 (1.1–3.2)	2.2 (1.5–3.2)	44/3/0/1
I (15)	120	25	1.2	0.5	12.0 (0.6)	29.1 (26.6–32.3) ^a^	2.8 (1.9–4.1)	3.3 (2.5–5.0) ^a^	13/2/0/0
B (12)	120	20 fixed	0.601	0.4	80.0 (0.625)	28.6 (25.1–31.6)	1.4 (fixed)	1.8 (1.6–2.1)	8/4/0/0
B (5)	120	20 fixed	0.601	0.4	80.0 (1.25)	29.9 (26.2–32.5) ^b^	1.4 (fixed)	1.7 (1.5–2.0) ^b^	3/2/0/0
N (6)	120	20	1.2	0.5	38.4	29.1 (24.1–31.8)	1.4 (1.3–1.9)	1.8 (1.5–2.3)	6/0/0/0
H (10)	120	*23 (NI)*	0.99	0.5	80.0 (0.625)	34.2 (29.8–37.9)	9.2 (6.5–12.2)	9.5 (7.2–11.3)	10/0/0/0
N (50)	110	51	0.6	0.23–0.24	57.6	29.8 (22.9–37.5)	1.7 (0.9–4.2)	2.1 (1.3–5.4)	45/5/0/0
A (7)	110	19	1.7	0.28	38.4 (0.6)	29.1 (26.5–31.7)	1.4 (1.0–2.1)	1.8 (1.3–2.7)	5/1/0/1
F (6)	100	124	0.758	0.5	80.0	31.7 (23.8–38.7)	6.2 (3.1–10.1)	6.8 (4.7–8.9)	5/1/0/0
K (24)	100	124	0.6	0.33	38.4 (0.6)	30.3 (25.1–34.6) ^c^	1.6 (0.9–2.1)	1.9 (1.3–2.5) ^c^	16/8/0/0
L (5)	100	75	1.2	0.3	38.4 (0.6)	30.9 (25.3–33.9)	7.4 (4.0–9.7)	8.6 (5.9–10.2)	3/2/0/0
E (60)	100	60	1.2	0.5	38.4	29.4 (24.5–38.6)	2.7 (1.5–6.6)	3.4 (1.8–6.0)	4/0/0/56
E (7)	100	60	0.984	0.7	40.0	31.4 (26.9–35.9)	2.5 (1.9–3.7)	2.9 (2.2–4.1)	0/0/0/7
J (57)	100	60	0.6	0.285	38.4 (0.6)	30.4 (21.4–40.5)	3.2 (1.6–7.5)	3.8 (2.3–6.6)	54/3/0/0
K (5)	100	*35 (NI)*	1.38	0.7	20.0 (1.25)	29.0 (27.8–29.9) ^d^	4.2 (2.1–5.9)	4.2 (2.7–5.8) ^d^	3/1/1/0
H (9)	100	*23.4 (NI)*	0.99	0.5	80.0 (0.625)	27.9 (24.8–29.6)	3.4 (2.7–3.9)	4.5 (3.5–5.3)	9/0/0/0
E (11)	Sn100 (DS)	200	1.7	0.285	38.4	29.5 (26.4–35.9)	1.9 (1.3–3.0)	2.4 (1.7–3.6)	1/0/0/10

n.e. = not evaluated; CTDI_vol_ = volumetric computed tomography dose index; DLP = dose length product; SSDE = size-specific dose estimate; TCTP = tube current–time product; NI = noise index; IQ = image quality. ^+^ Minimum and maximum TCTP with average value for adults in parentheses. Lateral diameters not measured in ^a^ 3 (site I), ^b^ 1 (site B), ^c^ 7 (site K), and ^d^ 2 (site K) patient(s).

## Data Availability

The underlying data cannot be made publicly available upon publication because they contain sensitive personal information. The data that support the findings of this study are available upon reasonable request from the authors.

## Supplementary Materials

The following supporting information can be downloaded at https://www.mdpi.com/article/10.3390/bioengineering11030207/s1. Table S1: Requested patient and scanner data from participating RACOON sites; Table S2: Requested subjective IQ parameters and CT major findings from participating RACOON sites; Table S3: Documented scanner types and vendors throughout the included cohort; Table S4: Protocol-specific mean CTDI_vol_ and anterior–posterior and lateral diameters with ranges per site corresponding to Table 4.
