# Supplementary material for: Standardization of a CT Protocol for Imaging Patients with Suspected COVID-19—A RACOON Project
Source: Bioengineering (Basel). 2024 Feb 22;11(3):207. doi: 10.3390/bioengineering11030207 (PMC10968361; doi:10.3390/bioengineering11030207)
Supplement: Supplementary file 1 [file bioengineering-11-00207-s001.zip › bioengineering-2855330-supplementary.pdf]

Supplementary Materials

Table S1: Requested patient and scanner data from participating RACOON sites.

Parameter	Unit
Patient diameter (center slice)	mm
$CTDI_{vol}$	mGy
DLP	mGycm
Scan coverage	cm
Effective tube potential	kV_p
Effective tube current – time product	mAs
Pitch	unitless
Collimation	mm
Reference settings (noise index or reference tube potential and reference tube current – time product, $TCTP_{ref}$)	variable (mAs, kV_p, noise index)
Automatic exposure control	enabled/disabled
Scanner information (vendor, model)	

Table S2: Requested subjective image quality parameters and CT major findings from participating RACOON sites.

Parameter	Value
Image quality	Good, suboptimal, insufficient
Reason for bad image quality	Motion, beam hardening, image noise, insufficient field of view
Intubation	No, tracheal cannula
Severity Score	1-18
COV-RADS	1-5
CO-RADS	1-6

Table S3: Documented scanner types and vendors throughout the included cohort.

**Vendor**	**Scanner**
GE	Brightspeed Elite
	Revolution
	Revolution CT
	Revolution Evo
	Revolution Frontier
Optima	CT660
Philips	Brilliance 16 P
	Brilliance Big Bore
	Brilliance iCT
	iCT 256
	Ingenuity CT
	IQon - Spectral CT
Siemens Healthineers	Biograph 40
	Biograph 20
	Biograph 6
	SOMATOM Drive
	SOMATOM Force
	SOMATOM Definition AS
	SOMATOM Definition AS +
	SOMATOM Definition Edge
	SOMATOM Edge Plus
	SOMATOM Definition Flash
	SOMATOM Go.Top
	SOMATOM X.cite
	Sensation Open
Toshiba	Aquilion

Table S4: Protocol-specific mean $CTDI_{vol}$ and anterior-posterior and lateral diameters with ranges per site corresponding to table 4.

**Site (included patients)**	**Diameter with range ap [cm]**	**Diameter with range lat [cm]**	**Mean $CTDI_{vol}$ with range [mGy]**	**Acquisitions with $TCTP_{eff}$ above $TCTP_{ref}$**	**Mean effective diameter [cm] of patients with $TCTP_{eff}$ < $TCTP_{ref}$ vs $TCTP_{eff}$ > $TCTP_{ref}$**
D (20)	27.2 (19.9-35.4)	37.6 (27.1-46.9)	10.7 (5.5-20.7)	12/20	27.5 vs 34.9
B (11)	23.5 (20.3-27.9)	33.4 (26.6-37.4)	3.7 (2.3-6.5)	3/11	28.0 vs 27.8
G (48)	25.0 (19.9-31.6)	37.3 (26.6-48.3)	1.9 (1.1-3.2)	17/48	28.5 vs 34.0
I (15)	23.8 (21.1-26.5)	35.4 (31.9-40.8)[a]	2.8 (1.9-4.1)	9/15	28.3 vs 29.9
B (12)	23.6 (19.2-27.4)	34.7 (28.7-40.9)	1.4 (fixed)	*	
B (5)	25.8 (20.4-28.4)	35.1 (30.9-38.6)[b]	1.4 (fixed)	*	
N (6)	25.7 (20.5-28.9)	33.1 (28.4-35.7)	1.4 (1.3-1.9)	6/6	29.1
H (10)	27.8 (24.1-32.7)	42.2 (35.1-48.1)	9.2 (6.5-12.2)	**	
N (50)	25.7 (18.7-32.7)	34.7 (27.9-46.3)	1.7 (0.9-4.2)	***	
A (7)	24.2 (19.8-27.6)	35.1 (30.2-39.5)	1.4 (1.0-2.1)	4/7	27.3 vs. 30.5
F (6)	27.6 (18.7-34.9)	36.6 (30.4-42.9)	6.2 (3.1-10.1)	4/6	25.7 vs 34.7
K (24)	25.3 (19.6-29.5)	35.3 (29.8-41.6)[c]	1.6 (0.9-2.1)	21/24	26.4 vs 31.1
L (5)	25.6 (20.3-28.5)	37.2 (31.6-41.0)	7.4 (4.0-9.7)	4/5	25.3 vs 32.2
E (60)	25.6 (18.8-36.9)	34.0 (28.3-47.0)	2.7 (1.5-6.6)	***	
E (7)	26.1 (22.4-29.5)	38.0 (32.3-46.5)	2.5 (2.2-3.7)	***	
J (57)	25.1 (17.6-41.3)	37.0 (24.7-52.8)	3.2 (1.6-7.5)	49/57	25.7 vs 31.1
K (5)	24.2 (22.8-25.8)	34.6 (33.3-36.6)[d]	4.2 (2.1-5.9)	***	
H (9)	22.4 (20.9-24.3)	34.8 (27.7-37.9)	3.4 (2.7-3.9)	**	
E (11)	25.0 (21.8-30.6)	34.9 (30.0-46.5)	1.9 (1.3-3.0)	***	

Abbreviations: n.e. = not evaluated, $CTDI_{vol}$ = volumetric computed tomography dose index, TCTP = tube current – time product. Lateral diameters not measured in [a]3 (site I), [b]1 (site B), [c]7 (site K) and [d]2 (site K) patient(s). * fixed TCTP, ** noise index employed, *** not evaluable.
